# Accelerated Cardiac Aging in Patients With Congenital Heart Disease

**DOI:** 10.3389/fcvm.2022.892861

**Published:** 2022-05-26

**Authors:** Dominga Iacobazzi, Valeria Vincenza Alvino, Massimo Caputo, Paolo Madeddu

**Affiliations:** Bristol Medical School, Faculty of Health Sciences, University of Bristol, Bristol, United Kingdom

**Keywords:** aging, congenital defect, proteostasis, inflammation, surgery, extracorporeal bypass

## Abstract

An increasing number of patients with congenital heart disease (CHD) survive into adulthood but develop long-term complications including heart failure (HF). Cellular senescence, classically defined as stable cell cycle arrest, is implicated in biological processes such as embryogenesis, wound healing, and aging. Senescent cells have a complex senescence-associated secretory phenotype (SASP), involving a range of pro-inflammatory factors with important paracrine and autocrine effects on cell and tissue biology. While senescence has been mainly considered as a cause of diseases in the adulthood, it may be also implicated in some of the poor outcomes seen in patients with complex CHD. We propose that patients with CHD suffer from multiple repeated stress from an early stage of the life, which wear out homeostatic mechanisms and cause premature cardiac aging, with this term referring to the time-related irreversible deterioration of the organ physiological functions and integrity. In this review article, we gathered evidence from the literature indicating that growing up with CHD leads to abnormal inflammatory response, loss of proteostasis, and precocious age in cardiac cells. Novel research on this topic may inspire new therapies preventing HF in adult CHD patients.

## Background

Congenital heart disease (CHD) is the most common type of birth defect, with a reported prevalence of 9 per 1,000 births. Heart failure (HF) represents an important cause of morbidity and mortality in patients with CHD (1–4). It occurs in ≈25% of adult CHD (ACHD) patients by the age of 30 and is not limited to severe cardiac defects (5–8). Individuals with lower-complexity malformations, which constitute the majority of ACHD, have a higher burden of adverse cardiovascular events relative to the general population, with hazard ratios ranging from 2 for coronary artery disease to 13 for HF (9). In the US, hospitalization for ACHD-related HF increased 91% from 1998 to 2011 and charges increased 258%, more than double that for non-ACHD HF.

Several factors may participate in the pathogenesis of ACHD-related HF, such as inherited architectural disorganization of myocytes and vascular cells, cardiac damage due to the surgical trauma, and insufficient protection during cardiopulmonary bypass, and the hemodynamic load to the heart from residual defects and failing grafts (10). Moreover, genetic and epigenetic factors play both distinct and additive roles in maladaptive myocardial remodeling (6). Investigation of mechanisms has mainly focused on canonical pathways, such as the renin-angiotensin-aldosterone system (RAS) and adrenergic system (11, 12). Nonetheless, trials using RAS inhibitors in ACHD-related HF failed to show any benefit on ventricular function (13, 14), thus conventional therapy is unsupported by clinical evidence (15). New efforts are urgently needed to unveil new druggable targets for prevention and treatment. Investigation in neonatal animal models could aid in this endeavor. Mice modeling monogenic causes of CHD recapitulate cardiac septation defects, heart valve malformations, and conotruncal heart defects, as well as more complex CHD (16, 17). More recently, patient-specific induced pluripotent stem cells (iPSCs) have been employed to study human CHD (18). In addition, large animal models simulating the corrective treatments employed in children are warranted to determine the positive and negative effects of cardiac surgery on post-natal heart maturation (19–22). Finally, computational growth models can help in tracking disease progression and stratifying patients with CHD (23, 24).

## Scope of This Review Article

Accelerated cardiac cell senescence is a key determinant of the increased risk for HF in patients with CHD. Hence, identifying the causes of heart’s aging may improve the outcome of CHD patients.

**FIGURE 1:**
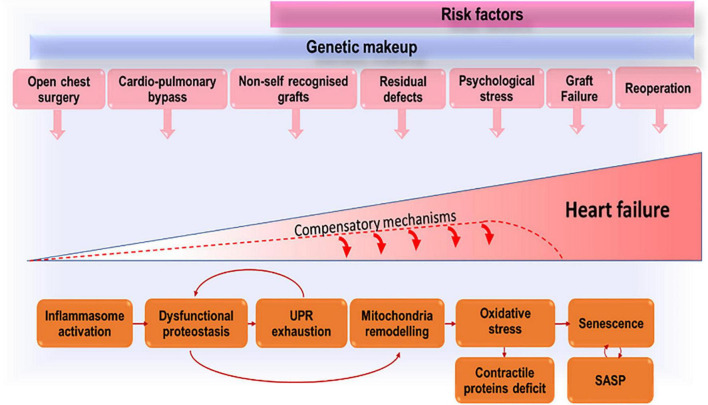
Accruing stressors that, by inducing premature cells senescence, lead to exhaustion of compensatory mechanisms and eventually heart failure.

We propose that growing up with CHD exposes the patient to multiple and repeated stress, related not only to the defect severity, but also to the damage from reconstructive and multiple cardiac operations, cardio-pulmonary bypass and cardioplegic arrest, and implantation of foreign graft material (Figure 1). Cardiac cells (especially stromal cells which have immunomodulatory roles) respond to early in life and subsequent repeated stress by activating the inflammasome. Stress-induced response pathways will attempt to reduce protein synthesis and increase the cellular capacity for protein folding and degradation. With unremitting stress, proteostasis will be overloaded, resulting in misfolded proteins accumulation in subcellular compartments, including the mitochondria where proteotoxic stress will incite ROS production. An increasing number of cells will become senescent in the CHD heart and will transfer proteostatic stress to neighboring cells through the secretion of inflammatory chemokines and misfolded peptides. This vicious cycle will lead to cardiomyocyte loss, microvascular rarefaction, and fibro-calcific interstitial remodeling of the heart, compromising myocardial perfusion and contractile function.

The stress from the defect and surgical trauma is unavoidable. However, drugs able to modulate the inflammatory response and to improve proteostasis could halt cardiac deterioration in CHD patients, thus succeeding where conventional therapy has failed. Moreover, improvement in graft biocompatibility, e.g., cellularization prior to implantation, may reduce the inflammatory reaction to the prosthesis and the need for reinterventions, therefore delaying heart’s aging and failure.

## Types and Etiology of Congenital Heart Disease

In a definition proposed by Mitchell et al. in 1971, Congenital Heart Disease is referred to as “*A gross structural abnormality of the heart or intrathoracic great vessels that is actually or potentially of functional significance*” (25). Since then, more than forty different types of defects have been characterized, from the most common ventricular septal defects (VSD), to the severe Tetralogy of Fallot (26).

The classification of CHD based on the anatomic complexity of the defect, from mild to moderate to complex, and on the patients’ related risk of morbidity and mortality is the most commonly applied system (27). However, in a more simplified stratification, CHD are normally divided into cyanotic and acyanotic (28, 29). Tetralogy Of Fallot (TOF) and Transposition of Great Arteries (TGA) are the two most common cyanotic CHD conditions, characterized by a right to left shunt resulting in deoxygenated blood entering the oxygenated limb of the vascular circuit, thus causing an oxygen saturation level below 90%. On the other hand, the majority of septal defects such as Ventricular Septal Defect (VSD), Atrial Septal Defect (ASD) and Atrioventricular Septal Defect (AVSD) are classified as acyanotic CHD lesions, with oxygen saturation normally above 95%.

Furthermore, CHD malformations can occur as single lesions or in combination with other heart defects. Commonly diagnosed CHD single lesions are ASD, VSD, and pulmonary stenosis (PS), whereas complex or combination lesions include AVSD, TOF, and TGA (30). Within this yet complex scenario, the mostly unknown etiology of CHD adds a further level of complexity to the CHD condition.

Although numerous etiologic investigations have been conducted, only approximately 15% of cases of CHD can be attributable to a known cause (31). Indeed, the etiology of CHD remains in most cases unknown. Numerous theories have been put forward to explain the pathogenesis of CHD, the earliest of which dates back 1968, whereby the author conducted and extensive and systematic investigation into the potential factors underlying the development of CHD (32). While the genetic basis alone, determined by either gross chromosomal aberration or single mutant genes, was considered and tested by reviewing karyotypes of CHD patients in the literature as well as 104 personal cases, this hypothesis was rejected for lack of conclusive evidence. Conversely, the author supported the hypothesis of the so called “*multifactorial inheritance*,” which encompasses both genetic and environmental factors as known to participate in the etiology of CHD.

Although the gene-environment interaction could certainly affect the normal embryo-fetal development, separate environmental and genetic causes have been also identified (33, 34).

### Genetic Basis

In 2007, the American Heart Association Congenital Cardiac Defects Committee conducted an extensive review on the contribution of genetics to the origin of CHD, with an insight on the available diagnostic genetic tools and their applications. Chromosome analysis and FISH technology were indicated as important tools to detect the chromosomal 22q11 deletion, which is associated with a series of CHD, including Tetralogy of Fallot, interrupted aortic arch type B, truncus arteriosus, cono-ventricular VSDs, and aortic arch anomalies (35, 36). Another common microdeletion, at chromosome 7q11.23, has been identified by FISH and associated to Williams-Beuren syndrome, an autosomal dominant disorder characterized by specific cardiovascular anomalies, including supravalvular aortic stenosis, often in conjunction with supravalvular pulmonary stenosis and peripheral pulmonary stenosis. The review also provides a list of single genes, whose mutation, thanks to the DNA Mutation Analysis technique, was found to be associated to a number of heart defects (i.e., GATA4 in ASD/VSD; CFC1 in TGA, FBN1 in Marfan syndrome, etc.) (33).

With the development of new genomic techniques, such as chromosomal microarray and next-generation sequencing (NGS), the discoveries of new gene mutations and numerous pathogenic copy number variants (CNVs) and have significantly advanced the understanding of causes of CHD (37). An updated summary of knowledge of the genetic contributions to the pathogenesis of CHD has been reported in 2018 by the same American Heart Association Congenital Cardiac Defects Committee that conducted the initial investigation on the genetic basis of heart defect in 2007 (38).

As more and more young patients with severe types of CHD are surviving into their adulthood, thanks to the advances in surgical strategies of intervention, ascertaining the genetic cause of the defect is an important determinant in providing more accurate prognosis for the CHD and outcomes for CHD–related interventions. Furthermore, determining whether there is an underlying genetic pattern to the heart defect can help evaluate associated extracardiac organ involvement and identify genetic reproductive risks for other family members.

### Environmental Risk Factors

Although genetic factors play an important role in the development of CHD, accounting for about the 20% of all defects, it has become increasingly evident that some CHD might be preventable through interventions addressed to reduce environmental exposure (39). Indeed, environmental factors are identified as the cause of approximately 5–10% of CHD, as estimated by the World Health Organization. Epigenetic changes during pregnancy can influence the development of child’s heart structure (40–42). Epigenetic mechanisms implicated in the pathogenesis of CHD include DNA methylation, histone modifications, higher-order chromatin structure, and the activity of non-coding RNA species reviewed in Lim et al. (43).

Epidemiological studies have suggested an association of CHD with various types of environmental factors to which pregnant women and their fetuses may be exposed, such as air pollutants, pesticides, solvents, metals, radiations, contaminants, and chemicals (44, 45). Lifestyle and socioeconomic determinant might constitute risk factors too (45, 46).

Among the main mechanism of action of exposure to chemical pollutant are DNA methylation, oxidative stress, inflammatory processes, and epigenetic mechanism (47–55).

It is conceivable that the impact of epigenetics is not restricted to the embryonic heart development, but the same responsible mechanisms could rather influence the aging clock of the congenitally defective heart.

### Accrual of the Aging Inducers in Patients Growing Older With Congenital Heart Disease

Advances in cardiac surgery and postoperative care during the past few decades have led to more people with CHD living longer with a better quality of life. Despite the progresses in this field, it is estimated that death rates in the population from 20 to > 70 years of age may be twice to seven times higher for the ACHD population than for their peers (56).

Residual hemodynamic abnormalities and new sequelae, after childhood surgical repair, might manifest later on in life, adding on to superimposed acquired heart disease and to the comorbidity of the natural aging process of the heart. It is now widely acknowledged that age represents the largest risk factor for cardiovascular disease, as cardiac aging entails a series of pathophysiological changes, impairing the myocardium at structural, cellular, molecular, and functional levels (57). These changes are highly relevant to cardiomyocytes, because they are postmitotic cells and thus not replenished by proliferation. Therefore, diminished myocardial reserves, due to a physiologic age-associated disruption of cardiac homeostasis, leads to increased vulnerability to cardiovascular diseases that occurs with aging.

Several environmental factors can contribute to accelerate the heart aging clock in ACHD people. Sedentary behavior and physical inactivity are among the leading modifiable risk factors worldwide for cardiovascular disease and all-cause mortality. Sedentary behavior is increasingly recognized as a risk factor for cardiovascular disease, independent of physical activity (58). The global childhood inactivity crisis is worrisome for the consequence on metabolic and cardiovascular disease (59). Children with CHD may be even less active than their healthy peers, although this evidence is matter of debate (60–63). In addition, very little is known about sedentary behavior in this population. A cross-sectional study with 316 children and adolescents with CHD seen in an outpatient clinic of a reference hospital showed the prevalence of excess weight was 26.9%, which is similar to the prevalence of that described in the literature for children without congenital disease (64).

Psychomotor development is influenced by factors such as low birth weight, cyanosis, hospitalizations, repeated examinations, physical constraints and consequently, school and social withdrawal. Children with CHD reportedly have a developmental delay (65). A larger study analyzing data from the 1997 to 2011 National Health Interview Survey showed that children with CHD are more likely to report worse health overall, to need more healthcare services, and to have other health conditions (e.g., autism, intellectual disability, or asthma), compared to children without CHD (66). The caregivers of children with CHD undergoing cardiac surgery are also under stress due to the uncertainty of the surgical outcome and the stressful experience of being admitted to an intensive care unit. The stress may influence the children perception of their state and amplify their frailty (67). Restriction in physical activity might be medically or parentally imposed. Furthermore, parents might overprotect their children in fear of sudden complications during physical exercise.

Growing to an adult with CHD exposes to an often traumatic transition from pediatric care to a different care model that lasts through a lifelong trajectory (68). Surprisingly, these concerns seem to be contradicted by a study that measured Quality of life (QoL) in patients with CHD (69). This observational case control study showed that patients with CHD felt their environment was healthier, had more opportunities for leisure and were happier with their access to healthcare system than controls. Except for the need for more medical treatment in patients with great CHD defects, no significant differences were seen in the World Health Organization QoL-BREF Index according to the anatomical complexity.

## Evidence of Age-Related Comorbidity

The recognition of an association of CHD with age-related comorbidity would strengthen the assumption of a more rapid senescence at the heart and systemic level. In geriatric ACHD patients, mortality is seemingly driven by acquired comorbid conditions. In a population-based cohort study, the most powerful predictors of mortality were dementia, gastrointestinal bleed, and chronic kidney disease (70). The increased risk for dementia, particularly early onset dementia, was confirmed by another cohort study using the medical registries and records from Danish hospitals between 1963 and 2012 (71). There was a 60% increased lifetime risk of dementia in individuals born with CHD compared to the public. In addition, even more alarming, there was a 160% higher chance of having dementia earlier than 65 years of age.

Coronary artery disease (CAD) is one of the most important causes for mortality in ACHD patients, and the consequences of an acute coronary event can be more fateful in a patient with a corrected congenital defect than in age-matched controls (72). The prevalence of CAD in a population of ACHD patients in the US was 6.8% (73). This cohort comprised ACHD patients who were symptom-free, one reason being that the heart might become denervated during an open-heart operation, as for example in patients whose transposition of the great arteries was corrected with arterial switch operation. The increased risk of CAD is not associated with a greater accrual of risk factors, with the only exception being arterial hypertension, whose prevalence is slightly higher and insurgence more precocious in patients with CHD (74). Moreover, a retrospective study suggested that metabolic syndrome was more common among adults with CHD than in the general population (75).

Interestingly, the literature indicates that patients with cyanotic CHD might be protected against atherosclerosis. This might be due to a combination of reduced atherosclerotic risk factors such as lower blood pressure, lower total cholesterol levels, higher bilirubin levels and lower thrombocyte levels (76–78). Another study argued that children and adolescents with cyanotic CHD have subclinical atherosclerosis as indicated by cross sectional assessment of carotid intima-media thickness (79).

Evidence of accelerated aging at the systemic level is supported by the observation that CHD patients suffer from sarcopenia, a condition characterized by progressive and generalized loss of skeletal muscle mass and strength (80, 81), osteoporosis (82), and have a twofold higher risk of developing cancer compared with healthy matched controls, with this risk being significantly higher among patients with CHD from the most recent birth cohort (83). The relative risk of cancer was high, regardless of whether patients had undergone surgical procedures or were exposed to diagnostic low dose ionizing radiation, which contradict the interpretation of another report from a different group (84). Despite these conflicting results, however, most research agrees that a senescent phenotype is observable in irradiated organs, in a process that starts with DNA damage induced by radiation, followed by G2 arrest and mitotic bypass. Ataxia telangiectasia-mutated protein, p53, and p21 are among the crucial mediators of the DNA damage response signaling network (85). The ionizing radiation-mediated cell senescence occurs in virtually all eukaryotic cells. Nonetheless, some cell types are more radioresistant than others. With regard to the heart, cardiomyocytes, which are terminally differentiated, quiescent cells, are highly radioresistant, whereas endothelial cell (EC) readily undergo senescence or permanent cell-cycle arrest after exposure to moderate dose of radiation (86, 87).

It was reported that activation of the insulin-like growth factor 1 (IGF1)-phosphtidylinositol-3-kinase (PI3K)-Akt/mechanistic target of mTOR pathway acts upstream of the p53-p21 pathway in EC senescence induced by radiation. Additionally, radiation-induced senescent EC exhibit decreased production of nitric oxide and elevated production of ROS, probably due in part to downregulation and/or upcoupling of endothelial nitric oxide synthase (eNOS) and upregulation of NADPH oxidases. The authors also report an increased expression of adhesion molecules and inflammatory cytokines, in addition to an inability to proliferate and form capillary-like structures *in vitro*, suggesting that EC senescence can lead to endothelial dysfunction by dysregulation of vasodilation and hemostasis, induction of oxidative stress and inflammation and inhibition of angiogenesis, which can potentially contribute to radiation-induced late effects leading to cardiovascular diseases.

## Mechanism of Cardiac Aging

Many of the age-related cardiac changes seem to be in common with the alterations underlying the development of the major cardiac pathologies, such as chronic HF, ventricular hypertrophy and atrial fibrillation. Highlighting the potential common pathways of heart aging and cardiac pathophysiology might provide an explanation of the age risk factor for these diseases and for the higher susceptibility of the ACHD population. The hallmark of cardiac aging is the functional impairment that manifests as systolic and diastolic dysfunction, as well as disturbance in the electrical activity of the heart, presenting with different types of arrhythmias (57, 88).

On a structural level, the most striking phenomenon seen with age is an increase in the thickness of the Left Ventricle (LV) wall because of increased cardiomyocyte size. LV hypertrophy is mostly seen as a compensatory response after the loss of cardiomyocytes with aging, and because of the increased afterload produced by large artery stiffening (89, 90). Similar anatomical changes, although not as prominent as in the left side, are observed in the right side of the heart, in conjunction with reduced end systolic and end diastolic volumes (ESV and EDV). Degenerative or calcific aortic valve disease is another common feature encountered among elderly patients, ranging from mild valve thickening without obstruction of blood flow, to severe calcification with impaired leaflet motion, or aortic stenosis (91).

At a cellular level, remodeling includes loss of cardiomyocytes, by either apoptosis/necrosis or autophagy, which induces a compensatory alterations of extracellular matrix (ECM) composition involving the synthesis of fibroblasts and the degradation of collagen through transforming growth factor-β (TGF-β) signaling (92). The resulting fibrotic status of the heart increases cardiac stiffness and reduces the cardiac compliance.

The above mentioned structural and functional changes are the direct consequences of the numerous changes that occur at a molecular level. Investigation of molecular mechanisms underlying cardiac aging has mainly focused on canonical pathways of neurohormonal signaling dysregulation, such as the renin-angiotensin-aldosterone system (RAAS) and adrenergic system (57, 88). Nonetheless, pharmacological intervention targeting these pathways did not show any benefit on ventricular function (13, 14).

Among the other molecular pathways associated with cardiac aging, the development of oxidative stress, because of excessive mitochondrial ROS generation and impaired antioxidant defense, has been extensively described. Elevated ROS levels in the aging myocardium lead to enhanced DNA and protein oxidation/nitration, activation of inflammatory response, apoptosis, and endoplasmic reticulum (ER) stress (93, 94). Moreover, the mitochondrial dysfunction, resulting from ROS-induced damage to mitochondrial DNA and proteins, impairs respiratory chain efficiency, leading to further ROS production in a vicious cycle that ultimately impacts on energy production and myocardial function (95). The excitation-contraction coupling also results affected by the oxidative damage, with the sarcoplasmic reticulum Ca2 + ATPase pump (SERCA) being a direct target of the ROS action, meaning a decreased pump activity and slow contraction in the aging heart (96).

Another important consequence of mitochondrial stress is the impairment of the proteostasis network, which plays a key role in safeguarding the functioning of the heart by maintaining proper cell function through adequate synthesis, folding, assembly, trafficking, function, and degradation of proteins (97, 98). Mitochondrial stress activates the mitochondrial unfolded protein response (UPR_mito_), which is regulated by activation of the transcription factor associated with stress 1 (ATFS1). The import of ATFS1 in the mitochondrion is prevented in stress situations, which results in ATFS1 being translocated to the nucleus instead, where it binds to the promotor region of genes involved in resolving mitochondrial stress (99). This derailed proteostatic network, in addition to other stress-responsive pathways [i.e., heat shock response (HSR), endoplasmic reticulum unfolded protein response (UPR_ER_)], and the aberrant protein degradation/autophagy activity, are common denominator in age and multiple cardiac diseases culminating in HF, suggesting that proteostasis maintenance is an important aspect of healthy cardiac aging, and therefore susceptible to potential pharmacologic intervention (99, 100).

More recently, miRNAs have been proposed as novel key players of cellular senescence regulators (101, 102). Among the several miRNAs differently expressed in the old vs. young mouse hearts, miRNA-22 overexpression was shown to be involved in aging-related cardiac fibrosis, while the aging-induced expression of miR-34 was associated to contractile dysfunction during age (103, 104). Therefore, investigating potential aging-related miRNAs and targeting them *via* pharmacological modulation could represent a strategic means to combat cardiovascular aging in a clinical setting.

Lastly, the reduced regenerative capacity of cardiac stem cell must not be ignored, as increasing evidence suggests (105–107). Such decline may in part be responsible for the impaired myocardial repair in aged hearts and, thus, enhancing the function of endogenous cardiac stem cells might represent a new promising therapeutic strategy of intervention in the senescent myocardium.

It should be noted that heart aging is not a homogenous process, as the changes with age do not occur in everyone at the same pace. The rate of aging myocardium is influenced by multiple factors, including genetic features, lifestyle, environmental exposure, and most importantly comorbidity.

## Aging Mechanisms in Congenital Heart Disease

Deeper research aimed at preventing the injury and pathophysiological changes that predispose the young CHD heart to premature aging are warranted. Furthermore, identifying markers of early senescence in ACHD is crucial to guide the development of novel therapeutic interventions.

Early hemodynamic overload represents the obvious mechanism making the heart prone to premature aging (108). Right ventricular (RV) failure can develop in young patients as consequence of the pressure overload such as in TOF, in which the RV becomes the systemic ventricle, and in Pulmonary Artery Hypertension (PAH) associated with left to right shunt. An overloaded RV undergoes a remodeling phase characterized by mitochondrial dysfunction, ischemic stress, and ROS overproduction.

### Senescence as a Determinant of Congenital Heart Disease Outcome and Irreversibility

RV failure in young CHD can be prevented by focused interventions, such as by reversing PAH through surgical correction of aortopulmonary shunts. However, this reversibility potential is lost beyond a certain point in time, and late correction leads to irreversible damage to the pulmonary vasculature resulting in chronic PAH development (i.e., Eisenmenger’s syndrome) (109). An interesting study has demonstrated how irreversible PAH-CHD is associated with a switch from a proliferative to a senescent vascular phenotype (110). In a shunt-induced PAH rat model, the authors found a significantly reduced level of genes associated with DNA repair (i.e., Rad51) and increased levels of survivin, a protein associated with apoptosis resistance, in irreversible vs. reversible PAH animals.

Senescence markers, such as p16^ink4A^ and p21^cip1^, and SASP were also upregulated in irreversible PAH compared to reversible PAH. The vascular profile of irreversible shunt-induced PAH in rats was further corroborated in human lung explant tissue from patients with irreversible PAH-CHD vs. controls, confirming markers of senescence (survivin, p16^ink4A^ and p21^cip1^) in human PAH-CHD tissue, and higher vulnerability to senescence of PAH-CHD tissue derived endothelial cells in response to shear stress. These observations have led the authors to the recommendation that PAH-CHD should be repaired in early infancy when PAH is still in at a reversible stage.

In the next sections, we will examine the mechanisms and pathways that lead to a progressively faster transition to irreversible aging of the congenitally defective heart.

### Inflammation

As previously reported, one of the main features of senescent cells is the secretion of SASPs, including soluble signaling factors, proteases, and insoluble proteins/extracellular matrix (ECM) components released into the surrounding cellular environment. Specifically, the secretome of senescent cardiomyocytes and vascular smooth muscle cells is rich in the inflammatory cytokines IL-1 and IL-6 and tumor necrosis factor a (TNF-α), inducing inflammation in the local surrounding cardiac microenvironment and systemically (111, 112).

Systemic chronic inflammation and immune activation are crucial determinant in the pathogenesis of various cardiovascular diseases and therefore regarded as potential therapeutic targets. Accumulating evidence indicates chronic inflammation and immune senescence have a pathogenic relevance in the progressive deterioration of heart function in patients with CHD (113).

#### Genetic Determinants Linking Congenital Heart Disease and Inflammatory—Immune Response

Although the inflammatory syndrome observed in CHD patients is generally described as a consequence of hemodynamic changes or postoperative response after corrective heart surgery, innate factors such as the host’s genotype might determine the magnitude of their systemic inflammatory status and the individual inflammatory response profile to stress factors (114, 115). In a study analyzing the systemic inflammation in new-borns with CHD undergoing heart surgery, it was found that serum IL12p70, IL-6, IL-8, IL-10, and TNF-α levels were significantly higher in CHD patients than in normal cord blood. In a similar study, Mou et al. provided direct evidence of myocardial inflammatory activation in children with CHD not only after CPB but even before surgery, when elevated pre-operative TNF-α levels and nuclear translocation of NF-KB were detected (116).

Additionally, genetic syndromes associated with CHDs can indirectly contribute to altered inflammatory and immune responses. In DiGeorge Syndrome, the abnormal development of pharyngeal precursor structure for both the heart and thymus results in cardiac defects and humoral and T-cell deficiency (117). Children with Down Syndrome are at higher risk for CHD, infections, and immune cell malignancies (118). In Turner Syndrome, congenital defects of bicuspid aortic valve and aortic damage are often associated with autoimmune disease. This immune connection is highlighted by the inflammatory remodeling of the bicuspid valves, which undergo premature immune-mediated calcification, and a systemic pro-inflammatory state (119).

#### Hemodynamic Influence on Inflammatory—Immune Response

There is a reciprocal augmentation between hemodynamic alterations (myocardial injury, hypoxia, edema, and hypoperfusion) and systemic and local myocardial inflammatory response mediated by circulating immune cells and cardiac macrophages (115, 120). Changes in the cytokine profile are acknowledged as part of a compensatory response to the hemodynamic stress in a variety of CHD, including septal defects and shunts. Cytokine response to mechanical stress encompasses high levels of necrosis factor (TNF)-a and acute-phase reactants (121, 122), which is intended to facilitate a compensatory structural remodeling (123).

Hypoxia induces the transcription of several inflammation-promoting genes, such as NF-κB and TLRs, *via* the alpha subunit of the hypoxia-inducible transcription factor (HIF-1α). Inflammatory cells and cytokines have been shown to be activated in the pulmonary microvasculature of patients with communications between cardiac chambers or the great arteries leading to increased pulmonary blood flow and pressure. A differential pattern of cytokines was described in children with high pulmonary vascular resistance compared to children with high pulmonary blood flow, with the first group showing higher levels of Macrophage migration inhibitory factor (MIF) and Interleukin 16 (124).

Reversal of flow through a septal defect in Eisenmenger syndrome is associated with a systemic proinflammatory response, with increased C-reactive protein (CRP) and Interferon (IFN)-γ levels (125). Elevation of hsCRP is considered an independent predictor of cardiovascular morbidity and all-cause mortality in patients with CHD (126). Furthermore, in children with coarctation of the aorta, increased inflammatory and apoptotic mediators, including IL-6, IL-10, TNF-a, and soluble Fas (sFas) may contribute to vascular disease as these children enter the ACHD stage (127).

Hypoperfusion and congestion of the bowel facilitate the translocation of bacterial endotoxins from the gut lumen into blood circulation (116, 120). Abnormalities in the inflammatory response and immaturity of the immune system predispose to infection by common pathogens, such as respiratory syncytial virus, and to increased risk of developing bronchopneumonia and sepsis. The readers are invited to consult an extensive review of the immune and inflammatory markers associated with CHD from Singampalli et al. (113). Most common features include reduced granulocyte activity against bacterial infections, T and B lymphocyte levels, naïve T-cell production and T-Cell Receptor Excision Circles (TREC) levels IgA and IgG levels, and complement levels, as well as increased suppressor T-cell function.

#### Surgery and Cardiopulmonary Bypass and Inflammation

Aging is a risk factor for cardiac surgery. The other way round, the trauma of cardiac surgery could accelerate the biological clock of the heart. ACHD patients undergo repeated corrective surgical procedures, cardiopulmonary bypass, and general anesthesia, which can influence the organ homeostasis in different manners.

Open-heart surgery will leave a highly noticeable scar along the area where the incision was made. These scars are the superficial equivalent of heart scars. Surgical correction of the cardiac defect requires cutting, suturing, and inserting a graft or a stent in the attempt to restore the physiologic anatomy and perfusion. Unfortunately, reparative corrections may need to be performed in subsequent stages to allow maturation of the infant or must be repeated because of graft failure. Recurrent damage can trigger an endless cycle of inflammatory responses, scarring, and repair. Cardiac scars are dynamic living structures. Surgical trauma, like other forms of cardiac injury, is followed by tissue necrosis, neutrophil infiltration, and macrophage-driven clearance of cellular debris. Infiltration of circulating and local macrophages occurs as early as a few hours post-injury. Next, the production of extracellular matrix proteins by fibroblasts creates the structural basis for a stabilized fibrillar scar. This whole process can take several weeks. The mature scar contains a spectrum of cells, is metabolically dynamic, and subjected to further spontaneous evolution, i.e., expansion and extension. Contractile properties of the scar rely on the presence of inflammatory cell, which persist in cardiac scars for many years following injury (128).

The healing of a surgically corrected cardiac defect is also dependent on the biocompatibility and integration of prosthetic grafts. Bioprosthetic xenografts, allografts, and synthetic materials have traditionally been used for correction of cardiac defects. However, none of these grafts has the capability to growth in accordance to the child’s growth. Prosthetic conduits and patches used for correction of cardiac defects are non-viable and are recognized as a foreign material by the host’s immune system (129). The consequent inflammatory reaction leads to fibrocalcific remodeling similar to that seen in atherosclerotic lesions (130).

Surgical access for corrective or palliative operations often involves the partial or complete removal of the thymus (131). Thymectomized CHD patients reportedly develop cancer, autoimmune diseases, and atopic diseases and have a higher risk for bacterial and viral infections compared with those without thymectomy (132). This increased risk has been attributed immunologic exhaustion, after early thymectomy (133, 134). A reduction in total and naïve CD4 + T helper and CD8 + cytotoxic T cell compartments reportedly persisted for 2 decades after thymectomy (135, 136). Furthermore, these patients have a shorter T cell telomere length compared to controls and showed a diminished T cell receptor repertoire with signs of oligoclonality as well as augmented memory T cell levels (136, 137). These are typical features of immune senescence as seen in the adaptive immune deficiency typical of the older general population.

Most neonates with congenital heart defects require surgery on cardiopulmonary bypass (CPB) in early infancy and multiple subsequent surgeries to correct residual defects or failing grafts. For instance, the hypoplastic left heart syndrome (HLHS), one of the most critical forms of CHD, requires an early palliative intervention, the Norwood operation, which is associated with a high mortality risk. Although these multiple corrections are necessary to prevent the development of HF, they are not free from complications and adverse effects.

There is a consensus that a significant proportion of morbidity is related to the non-physiologically nature of total CPB, which activates an inflammatory response that closely resembles the systemic inflammatory response syndrome (SIRS), a condition characterized by cardiovascular and pulmonary dysfunction, coagulopathy, and multisystem organ dysfunction/failure (138). The reintroduction of high oxygen level following CPB exposes to reoxygenation injury is considered the primary trigger of the adverse inflammatory response (139, 140). Heterogeneity in the magnitude of the inflammatory response to reoxygenation may be attributed to genetic predisposition and severity of the cardiac defect. For instance, cyanotic CHD children suffering from chronic hypoxia are more prone to the reoxygenation injury during and after CPB, due to the reduced antioxidant reserve capacity in hypoxic conditions (141, 142).

Seminal transcriptomic and proteomic studies have attempted to dissect the complexity of the inflammatory pathways (143, 144). A recent investigation explored the whole blood transcriptome profile in neonates with HLHS before and after their Norwood operation, attempting to unveil an association with the development of the post-operative cardiac output syndrome (LCOS). There were distinctive differences in gene expression between patients with or without LCOS both in pre-operative (14 genes) and post-operative samples (8 genes). Moreover, those who developed LCOS showed larger differences between post-operative and pre-operative samples. Pathway analysis revealed differential regulation of inflammatory pathways (IL signaling, PDGF, NOTCH1, NGF, GPCR) and metabolic pathways (heme metabolism, oxidative phosphorylation, protein metabolism including amino acid and derivatives, fatty acid metabolism, TCA cycle and respiratory electron transport chain). The authors concluded that transcriptome profiling could provide diagnostic hints and potential therapeutic targets to improve outcomes in this high-risk population (114).

Moreover, CPB predisposes to postoperative infections, which impact on the postoperative rehabilitation of children with CHD. This predisposition has been attributed to suppression of the Toll-like receptor (TLR)-mediated signal transduction pathway, which compromises the inflammatory response (145). The same group showed that CPB suppresses the expression of the nucleotide binding and oligomerization domain (NOD)-like receptors signaling-mediated inflammatory response in pediatric CHD patients (146). NOD1 and NOD2 belong to the large class of Pattern recognition receptors which act as sensors of different ligands of bacterial origin. They both recruit and interact with the adaptor protein receptor-interacting protein kinase 2 (RIP2) and thereby activate the NF-κB pathway and mitogen-activated protein kinase (MAPK) pathway, which eventually induces the production of inflammatory cytokines, such as TNF-α and IL-6. Interestingly, peripheral blood leukocytes from CHD patients release TNF-α and IL-6 after stimulation with a NOD agonist, but this response is reduced after CPB (146). Altogether, these findings indicate that the injury from reperfusion causes a dysregulation of the innate immune response, resulting in susceptibility to infection and protracted low-grade inflammation.

### Inflammation as Therapeutic Target

Anti-inflammatory treatment, immunomodulatory drugs, and refinement of surgery to avoid or limit the need for thymectomy should be considered to reduce the damage caused by chronic inflammation and immune senescence. Translational work involving scar-modifying treatments has mainly focused on myocardial infarction (MI), with the main aims being to develop interventions that will steer scar properties toward compaction, mechanical strength, and electrical integration. Besides conventional drug treatments, more recent attempts to influence fibroblast activation involve approaches modulating the production of extracellular matrix protein, blocking inflammatory mediators, and interfering with TGF-β or Smad3 signaling (147–149). The effectiveness and safety of such interventions in children with CHD remains unknown. More advanced is the translational attempt to improve the biocompatibility of prosthetic grafts by tissue engineering, which, by combining the use of autologous/allogenic cells and biocompatible scaffolds, holds the promise to create cardiovascular grafts with the potential to remodel, repair, and grow alongside the children’s growth. In particular, the immune-favorable and immune-privileged status of stem/progenitor cells has made these cell types the ideal candidates for correction of congenital pathologies through a tissue engineering approach (150–153). Moreover, pharmacological modulation of altered pathways could be applied to reduce autologous stem cells senescence *ex vivo*, in order to enhance their reparative ability upon implantation (154). The readers are directed to recent reviews and preclinical studies on this topic (19, 20, 133, 155, 156).

In the last decade, different strategies have been proposed with the aim of avoiding reoxygenation injury during and after CPB. A study comparing propofol combined with low dose fentanyl and midazolam showed that the former is superior in reducing inflammation and oxidase stress and in improving post-operation recovery in children with CHD undergoing cardiac surgery (157). The use of controlled reoxygenation using a partial pressure of oxygen in arterial blood (PaO2), similar to the patient’s preoperative oxygen saturation, has shown to decrease the markers of organ damage (troponin I), stress (cortisol), inflammation (IL-6, IL-8, IL10) and oxidative stress (8-isoprostane) in single-ventricle and TOF patients undergoing cardiac surgery (158, 159). Other studies in adults and small randomized controlled trials in children have suggested some benefits (i.e., reduction of oxidative stress injury) in keeping the blood at normal body temperature throughout surgery (“normothermia”) as opposed to the standard hypotermic procedure, whereby the blood is cooled down during the operation (160, 161).

Senescent vascular cells can drive an irreversible state of vasoconstriction through a paracrine inflammatory mechanism. Therefore, their eradication could be an effective modality to halt the progression of PAH. Human pulmonary endothelial cells of patients with PAH are more vulnerable to senescence than controls in response to shear stress and the senolytic ABT263 reportedly induced apoptosis in senescent, but not in normal, endothelial cells. Interestingly, the senolytic treatment reversed the hemodynamic and structural changes associated with severe PAH in a rodent model (110). The pros and cons of senolytic therapy as a treatment of cardiovascular disease has been recently reviewed (162), although evidence supporting a benefit in children with CHD is lacking. The possible side effects on the young heart are also unknown.

Vitamin supplementation has been considered as a possible avenue for the primary prevention and treatment of cardiac defects. Peri-conceptional multivitamin use was associated with a reduced risk for CHD, particularly outflow tract defects and ventricular septal defects, but no risk reduction was evident when multivitamin use was started after the first month of pregnancy (163). Later studies focusing on folic acid showed that lower dietary folate intake during pregnancy was associated with increased risk (164). Moreover, maternal folate supplementation was significantly associated with a decreased risk of CHD, with this effect being possibly ascribed to anti-apoptotic protection of neural crest cells which participate in the formation of the truncus arteriosus and its division into the pulmonary artery and aorta (165, 166). However, another large study conducted in cohorts from Denmark and Norway found no association between individual-level of maternal folic acid supplementation and offspring heart defects. The authors concluded that, although most likely not harmful, the preventive effect of maternal folic acid supplementation indicated by time trend analyses should be questioned, at least in regions with sufficient intake of dietary folate (167).

Several studies reported that up to 90% of children with CHD undergoing corrective surgery have post-operative serum vitamin D concentrations below 50 nmol/L (168–170). Importantly, there was statistically significant direct correlation between serum vitamin D concentrations and post-operative cardiovascular dysfunction (171). The abrupt decline in vitamin D concentrations during surgery coincided with the initiation of cardiopulmonary bypass (170, 171). Usual low-dose daily supplementation is inadequate to maintain post-operative vitamin D concentrations above 50 nmol/L in the CHD population (172).

### Endoplasmic Reticulum Stress

As previously mentioned, the oxidative stress has been identified as one of the major contributors to the cardiovascular aging process. Elevated oxidative stress in the senescent myocardium has several consequences such as enhanced protein oxidation/nitration, activation of inflammatory response, proteostasis impairment, mitochondrial dysfunction and endoplasmic reticulum (ER) stress (88).

The ER plays key roles in the synthesis, folding, and translocation of secretory and membrane proteins and in calcium homeostasis and lipid biosynthesis (173). A variety of environmental stresses, including nutrient deprivation, hypoxia, calcium depletion, and cytotoxic drug administration, can cause the accumulation of unfolded proteins in the ER, thereby inducing the ER stress response. In the developing heart, an excessive or too prolonged activation of the UPR may reduce expression of essential proteins, adversely affect cell function, and eventually lead to cell death.

Interestingly, a recent study showed that exposure of mouse embryos to short-term gestational hypoxia induced the UPR in cardiac progenitor cells and caused common types of heart defect in about 50% of newborns (174). One of the early UPR responses was PERK-dependent attenuation of protein synthesis, which was expected to result in the reduction in embryo growth rate. However, in mouse embryos exposed to hypoxia, the translation block was only affecting the levels of specific proteins that are immediately and continuously available for cardiomyocyte maturation. In particular, gestational stress resulted in reduced FGFR1 translation, which profoundly reduced FGF signaling in cardiac progenitor cells of the second heart field, whereas other receptor tyrosine kinases characterized by rapid recycling were not affected (174).

In the above mouse model, the induction of the UPR was reversible but FGF-1 signaling was persistently abrogated (174). However, it is tempting to speculate that persistent hypoxia after birth may cause a progressive exhaustion of the UPR, leading to accumulation of unfolded proteins in the ER and mitochondria. A study from Jian et al. examined this possibility on myocardial samples from patients with cyanotic congenital cardiac defects (175). They found that the upregulation of HIF-1α in the hypoxic myocardium was associated with a marked induction of the chaperone GRP78/Bip in cardiomyocytes, suggesting an activation of the UPR. Then, they assessed the activity of the key signaling pathways of UPR, ATF6α, and PERK, and found that, at variance with the mouse embryo hypoxia model, ATF6α was significantly upregulated whereas ERK signaling was not significantly different between the cyanotic and acyanotic groups. This was interpreted as a compensatory mechanism, as pharmacological inhibition or silencing of ATF6α promoted cardiomyocyte apoptosis following long-lasting *in vitro* hypoxia or hypoxia/re-oxygenation (175).

The regulation of UPR under hypoxia may involve a group of hypoxia-microRNAs. For instance, miR-199a-5p is reportedly downregulated in hypoxic myocardial samples from children with CHD (139). This is mediated by the activation of IL-6/IL-11/STAT3 signaling. Binding of pSTAT3 to the promoter region of miR-199a-2 gene, more significantly under hypoxic conditions, reduced the expression of pri-miR-199a-2, a precursor of miR-199a-5p, thereby activating its UPR targets, GRP78 and ATF6 (176, 177).

### Dysfunctional Proteostasis

Recent findings indicate that cardiac aging is accompanied by a gradual derailment of proteostasis, which predisposes the heart to the development of age-related cardiac diseases, including atrial fibrillation (99).

Unfolded proteins can accumulate in cardiomyocytes due to genetic mutations, such as in desmin-related cardiomyopathy, but also as the consequence of cellular stress that damages nascent or aging proteins, or normal wear and tear. When terminally misfolded proteins exceed the UPR capacity, they are targeted for degradation through the ubiquitin–proteasome or autophagy–lysosomal pathways. Eventually, accumulation of misfolded proteins in aggresomes occurs when protein misfolding is not fixed. It is well acknowledged that the functionality of these regulatory proteostasis checkpoints is damaged during aging, with senescent cells becoming unable to maintain proteostasis and causing the heart to suffer from age-associated proteinopathies (178).

Ubiquitin plays a key role in the degradation of unfolded or misfolded proteins by targeting ubiquitin chains in the ubiquitin–proteasome or autophagy system. Failure to eliminate the abnormal protein aggregates causes the cells to move to apoptosis and death. SUMOylation is an essential component of the ubiquitination proteasome system. SUMO (Small ubiquitin-like modifier) conjugation pathway is abundantly expressed in the heart and implicated in cardiovascular development *via* modifying transcription factors necessary for normal cardiovascular development (179). A balanced post-translational SUMO conjugation-deconjugation pathway is essential for normal cardiac development. In fact, both hetero- and homo-zygous SUMO-1 knockout mice exhibited ventricular defects with high mortality rates, which were rescued by cardiac re-expression of the SUMO-1 transgene (179). Moreover, transgenic mice with cardiac-specific expression of SENP2, a SUMO-specific protease that deconjugates sumoylated proteins, resulted in premature death of mice due to atrial and/or ventricular septal defects. Surviving mice showed growth retardation, and developed cardiomyopathy with impaired cardiac function with aging (180).

Lysosomes are one of the main components of the autophagy pathway, which is crucial for maintaining cardiomyocyte morphology and function by regulating organelle turnover and cardiac remodeling. Alterations of mitochondrial function can impair processes such as cardiomyocyte differentiation, response to ischemic events, apoptosis, and autophagy (181). Mitophagy, the degradation of mitochondria by the autophagy machinery, is dysfunctional in primary human derived from subject with Down Syndrome and this is associated with low Parkin and p62 levels and delayed PINK1 activation (182). Moreover, mTOR hyperactivity and reduced ATG proteins involved in autophagosome formation contribute to downregulation of mitophagy (182). Organelle dysfunction could contribute to accelerated aging in patients with Down Syndrome-related CHD (183).

### Therapies Targeting Endoplasmic Reticulum Stress and Proteostasis

Therapies specifically targeting the ER stress and proteostasis in heart disease are at experimental stage. Some pharmacological agents used in clinical settings such as angiotensin II type 1 receptor antagonists and the antidiabetic agent pioglitazone may inhibit UPR pathways. Pharmaceutical AMPK activators reduce cardiac ER stress and prevents the progression of HF (173). Additional drugs include small-molecule activators of the heat shock response, such as geranylgeranylacetone, currently used in Japan for treatment of ulcers, and UPR_ER_ activators, such as the ATF6 pathway activator compound (184). The rationale for the use of these compounds is to activate stress response pathways to enhance cytoprotection against protein misfolding and aggregation. However, strategies to reduce protein synthesis could negatively interfere with regenerative processes. In contrast, the use of chemical chaperones such as 4-phenylbutyrate or agents that reduce misfolded protein concentrations may represent a more effective modality in CHD, where the UPR is exhausted and protein misfolding accrues slowly over time. In these conditions, activation of autophagy (e.g., rapamycin or spermidine), pharmacological proteasome induction, and interfering RNA–based therapeutics represent additional promising options (185). On the other hand, inhibition of autophagy might represent a target for treatment of some cardiovascular diseases which are not caused by the overexpression of a mutant protein, and are rather associated to excessive activation of autophagy, like myocardial infarction, and atrial fibrillation (99). Nonetheless, inhibitors of autophagy, such as bafilomycin, can cause cellular toxicity (186).

Moreover, longevity genes and proteins could have a significant impact in delaying age-related cardiac dysfunction (187). In this respect, we discovered that a genetic polymorphism of the BPIFB4 gene is associated with exceptional longevity. The related mutant protein forms a complex with 14-3-3 and HSP90, activating the prosurvival eNOS—Akt signaling. Supplementation of the longevity genes significantly improved cardiac function in different models of age-related cardiac disease (188–191) and reverse immune senescence (192). It would be interesting to determine the expression of BPIFB4 in CHD and, if this is found downregulated as in elderly cardiovascular patients (193), to assess the benefit of supplementation on cardiac cells from CHD patients and CHD models.

Personalized interventions are urgently needed for the treatment of rare cardiac diseases characterized by an alteration in protein disposal. For instance, the Koolen-de Vries syndrome (KdVS) is a rare monogenic disorder characterized by intellectual disability, heart failure, hypotonia, and congenital malformations. Mechanistic study shows that KANSL1 modulates autophagosome-lysosome fusion for cargo degradation *via* transcriptional regulation of autophagosomal gene, STX17. Kansl1 ± mice have an impaired autophagic clearance of damaged mitochondria and tend to accumulate ROS which in turn cause defective neuronal and cardiac functions. An FDA-approved drug, 13-cis retinoic acid, can reverse these mitophagic defects and neurobehavioral abnormalities in Kansl1 ± mice by promoting autophagosome-lysosome fusion. Hence, KANSL1 could represent a possible therapeutic target to treat autophagy and KdVS (194).

### Mitochorial Dysfunction

With the heart being a highly metabolic organ that is reliant on the maintenance of cellular-energetic homeostasis, mitochondria are important determinants of cellular longevity. It is, therefore, not surprising that defects in mitochondrial bioenergetics have been related to normal cardiac aging (195–197). Many factors contribute to the age-related reduced energetic capacity of the cardiac mitochondria including increased ROS, mutation and deletions in the mitochondrial genome, and dysregulation of proteostasis and mitochondrial biogenesis throughout the organism lifespan as well as in the embryonic development (95, 198).

Accruing evidence suggests that mitochondria maturation and processing not only serve as the powerhouse enabling the heart to beat but also play a critical role in regulating embryonic and neonatal heart development in mammals (199). The normal assembly of respiratory chain components, i.e., the oxidative phosphorylation (OXPHOS) complexes, in embryonic hearts is paramount to prepare the heart to undergo the fetal to neonatal metabolic shift -from anaerobic glycolysis to aerobic oxidative phosphorylation- after birth (199). Several studies have reported how defective OXPHOS in embryonic hearts leads to severe heart defects and embryonic or neonatal lethality in mouse models and human patients (200–202).

Additionally, a mismatch between mitochondrial programming and the substrate availability was proposed as the major cause for the juvenile cardiomyopathy described in mice with cardiac ectopic expression of MFN2 AA (203). Another *in vivo* study showed that cardiac ablation of both peroxisome proliferator-activated receptor gamma coactivator 1-α (*Ppargc1*α) and peroxisome proliferator-activated receptor gamma coactivator 1-β (*Ppargc1*β), nuclear transcriptional coactivators required for mitochondrial biogenesis, caused mitochondrial dysfunction and late gestational defects in cardiac function and cardiomyocytes maturation (204).

Strong evidence indicates that mitochondrial dysfunction, in particular impaired mitochondrial biogenesis (highlighted by decreased mitochondrial mass and DNA content), participates in the development of HF (205–207). Myocardial ischemia, reperfusion, and chronic overload significantly damage the mitochondrial structure and function, severely compromising cellular viability. A recent study showed reduced mtDNA replication and depletion of mtDNA together with pathological changes of mitochondrial ultrastructure occurs early during the development of RV hypertrophy, preceding the clinical manifestation of HF (208). The reasons for these alterations remain unknown but the authors suggested that “*these findings provide an important basis for the future development of mitochondrial oriented clinical and surgical treatment of patients with overloaded RV caused by CHD”* (208).

As previously described in the mechanisms of cardiac aging, mitochondrial dysfunction leads to elevate ROS production also in fetal heart, an event that triggers the DNA damage pathway that blocks cardiomyocyte proliferation (199). A study conducted on TOF patients with RV hypertrophy showed an increase of 4-HNE on mitochondrial function and structure. This bioproduct of lipid peroxidation, a form of oxidative stress in aging heart, altered the energy production in the mitochondrion of RV leading to its failure and cardiomyocyte dysfunction (209). An *in vivo* genetic model of mitochondrial dysfunction has been achieved by inactivating the mitochondrial transcription factor A (*tfam*), which resulted in elevated ROS production and activated DNA damage response, thus causing cardiomyocyte cell cycle arrest and ultimately lethal cardiomyopathy (210). The authors further showed that inhibition of ROS or the DNA damage response pathway rescued the cell proliferation defect observed in cultured fetal cardiomyocytes in which Tfam was deleted, making these pathways potential targets of intervention to prevent cardiac aberration caused by some forms of mitochondrial dysfunction.

Genetic factors, like mtDNA mutations, might as well underlie the mitochondrial stress typical of some heart disease, as we have previously described in the etiology of CHD (211–213). A Chinese study conducted a systematic screening assessing mtDNA mutations among coronary heart disease patients (214). One three-generation family presenting evidence of coronary heart disease maternally inherited was identified, with 6/24 analyzed adults in this family exhibiting coronary heart disease of vary severity. The sequencing of the mitochondrial genomes of these individuals showed a tRNA^Thr^ 15910C > T mutation of the M7b’c haplogroup, which is predicted to destabilize the strongly conserved (24C-10G) base-pairing, thereby disrupting tRNA^Thr^ functionality. Interestingly, only limited changes in mitochondrial functionality were observed, suggesting that 15910C > T mutation is relatively mild and may by itself be insufficient to produce a clinical phenotype, nonetheless it represents an inherited risk factor for the development of coronary heart disease. This finding points out how the diagnostic process of mitochondrial disease of the heart can be very challenging, especially in CHD patients, as it goes asymptomatic in the early stages, and later it manifests itself as cardiomyopathy, HF, arrhythmia and so on (215–217).

Identifying early markers of mitochondrial dysfunction would be beneficial to prevent the later manifestations of cardiac disorders in CHD population. It has been suggested that mitochondrial respiration in peripheral blood mononuclear cells (PBMCs) may have utility for predicting HF risk in some CHD, as elevated PBMCs respiration was found to be correlated with HF in post-Fontan SV-CHD young patients (218).

### Therapies Targeting Mitochondrial Dysfunction

Current treatments are not effective in preserving mitochondrial structure and function and therefore accruing mitochondria dysfunction through repeated operations could rapidly lead to energetic deficits in cardiomyocytes of CHD patients. A novel therapy based on mitochondrial transplantation may become a game-changer in this clinical field. Autologous mitochondria isolated from the patient’s own body and then directly injected into the ischemic myocardium significantly ameliorated ischemia/reperfusion injury and enhanced myocardial cellular viability and post-ischemic functional recovery (219). The authors reported that improvement in energy generation were associated with upregulation cardioprotective cytokines. The initial uncontrolled clinical application in humans was performed in pediatric patients suffering from myocardial ischemia reperfusion injury following coronary artery occlusion and revascularization (220). In a subsequent pilot study, mitochondrial transplantation enhanced ventricular strain in patients requiring postcardiotomy extracorporeal membrane oxygenation for severe refractory cardiogenic shock after ischemia-reperfusion injury (221).

## Clinical Implications and Future Directions of Aging-Related Congenital Heart Disease

The successful diagnosis, advances in imaging, catheter-based interventions, surgery, and clinical management of CHD have yielded a dramatic change in age distribution of the CHD population. The improvement in survival has increased the number of ACHD and related complications. Comparing ACHD group with the general population, the mortality of ACHD is drastically high (56). Results from the CONCOR study in Netherlands, showed that ∼ 0.6% of ACHD patients died because of the presence of late complications for cardiac causes. The 26% died for chronic HF and 19% died for unknown sudden death. Among the 23% who died for non-cardiovascular causes, 14% died for pneumonia, and 9% died for malignant diseases (222). Growing up with CHD implore clinicians and researchers to gain a better understanding of aging, as this will be the keystone to how this rapidly evolving group of patients will be planned and monitored (223). There is an urgent need to standardize a clinical-educational program in the transition phase between adolescents and adults to allow young patients to become more skilled about their disease and reducing the probability of delaying CHD care (diagnosis, complication assessment, therapy) in adulthood (224). Patients must have more awareness of their CHD conditions and take the responsibility of their decisions of care, such as, major interventions or transplantation (225). American College of Cardiology (ACC) and American Heart Association (AHA) member committee in 2018 have provided clinical guidelines with all the recommendations for the management of adult patients with CHD (226). This guideline is a complete revision of previous 2008 ACC/AHA guidelines and focuses only on the care of ACHD. This document was set up because of the successful diagnosis and treatment strategies employed in babies and children with this disease which have led to a high prevalence of the adult population. ACHD management requires specific needs, and cardiologists must be specifically trained for their care in highly specialized ACHD centers. Recently European Society of Cardiology (ESC) working group published a position paper with the recommendation for the transcatheter intervention in ACHD highlighting the issues of its allocation on national scale and how to train the surgeons to reach a standard recognition as ACHD interventionists (227). This process is running at slow pace because it will require infrastructures and expertise of different cardiologists for each procedure enabling the training of the future ACHD interventionists. In addition, stem cell therapy, immunosuppressive therapy for cardiac transplantation, mechanical pumps, artificial hearts and new machines using algorithms to estimate prognosis and guide therapies in ACHD patients will be one of the new interesting clinical research in the field of CHD (225, 228).

Beyond clinical and educational research program and specialized ACHD centers, several measures of aging underpinning ACHD will need to be studied. Scientific data on functional and biological markers of aging (telomere length, markers of inflammation, epigenetic clock) in CHD is hugely needed. For instance, exercise and diet could be potential strategies for maintaining the telomere. In addition, epigenetic modifications such as, DNA methylation, histone modifications, non-coding RNA, can be chemically reversibile and used as target for rejuvenation strategies (223, 229). Once aging is investigated over the entire life course and the predictor factors causing accelerated senescence in ACHD are characterized, a targeted therapy could be developed and applied to these patients. Furthermore, it is paramount to secure more resources which welcome patients in the field of CHD management and ensure a complete consciousness and responsibility of conducting a healthy life (225).

## Conclusive Remarks

The increase in lifespan of CHD patients poses new challenges to the National Health Systems and the society, but also provides an opportunity to increase translational efforts to design an holistic approach for the prevention and cure of age-related diseases. As for older people, it is vital to prolong the period ACHD patients enjoy a healthy life. Further refinement of surgical approaches including the merging of minimally invasive surgery and tissue engineering will limit the need of repeated operations. Anti-aging treatments targeting inflammatory pathways, and homeostatic response to stress merit consideration. Senolytic drugs provide a promising means to interrupt the vicious cycle of cell senescence and inflammation. Finally, genetic interventions transferring longevity genes and proteins could confer cardiovascular protection as shown in preclinical models of aging.

## Author Contributions

PM was responsible as corresponding author for the final writing and the data presented. All authors read, approved the final manuscript, conceived, and designed the review article.

## Conflict of Interest

The authors declare that the research was conducted in the absence of any commercial or financial relationships that could be construed as a potential conflict of interest.

## Publisher’s Note

All claims expressed in this article are solely those of the authors and do not necessarily represent those of their affiliated organizations, or those of the publisher, the editors and the reviewers. Any product that may be evaluated in this article, or claim that may be made by its manufacturer, is not guaranteed or endorsed by the publisher.
