# Corrigendum: Accelerated cardiac aging in patients with congenital heart disease

**DOI:** 10.3389/fcvm.2022.1112510

**Published:** 2022-12-15

**Authors:** Dominga Iacobazzi, Valeria Vincenza Alvino, Massimo Caputo, Paolo Madeddu

**Affiliations:** Bristol Medical School, Faculty of Health Sciences, University of Bristol, Bristol, United Kingdom

**Keywords:** aging, congenital defect, proteostasis, inflammation, surgery, extracorporeal bypass

In the published article, there was an error in the **Funding** statement. The BHF Funding grant number was missing. The correct **Funding** statement appears below.

